# The Use of a Combination of *alkB* Primers to Better Characterize the Distribution of Alkane-Degrading Bacteria

**DOI:** 10.1371/journal.pone.0066565

**Published:** 2013-06-18

**Authors:** Diogo Jurelevicius, Vanessa Marques Alvarez, Raquel Peixoto, Alexandre S. Rosado, Lucy Seldin

**Affiliations:** 1 Laboratório de Genética Microbiana, Departamento de Microbiologia Geral, Instituto de Microbiologia *Paulo de Góes*, Universidade Federal do Rio de Janeiro, Rio de Janeiro, Brazil; 2 Laboratório de Ecologia Molecular Microbiana, Departamento de Microbiologia Geral, Instituto de Microbiologia *Paulo de Góes*, Universidade Federal do Rio de Janeiro, Rio de Janeiro, Brazil; Missouri University of Science and Technology, United States of America

## Abstract

The alkane monooxygenase AlkB, which is encoded by the *alkB* gene, is a key enzyme involved in bacterial alkane degradation. To study the *alkB* gene within bacterial communities, researchers need to be aware of the variations in *alkB* nucleotide sequences; a failure to consider the sequence variations results in the low representation of the diversity and richness of alkane-degrading bacteria. To minimize this shortcoming, the use of a combination of three *alkB*-targeting primers to enhance the detection of the *alkB* gene in previously isolated alkane-degrading bacteria was proposed. Using this approach, *alkB*-related PCR products were detected in 79% of the strains tested. Furthermore, the chosen set of primers was used to study *alkB* richness and diversity in different soils sampled in Carmópolis, Brazil and King George Island, Antarctica. The DNA extracted from the different soils was PCR amplified with each set of *alkB*-targeting primers, and clone libraries were constructed, sequenced and analyzed. A total of 255 *alkB* phylotypes were detected. Venn diagram analyses revealed that only low numbers of *alkB* phylotypes were shared among the different libraries derived from each primer pair. Therefore, the combination of three *alkB*-targeting primers enhanced the richness of *alkB* phylotypes detected in the different soils by 45% to 139%, when compared to the use of a single *alkB*-targeting primer. In addition, a dendrogram analysis and beta diversity comparison of the *alkB* composition showed that each of the sampling sites studied had a particular set of alkane-degrading bacteria. The use of a combination of *alkB* primers was an efficient strategy for enhancing the detection of the *alkB* gene in cultivable bacteria and for better characterizing the distribution of alkane-degrading bacteria in different soil environments.

## Introduction

Alkane-utilizing bacteria are widespread in marine and terrestrial environments [1], [2]. These bacteria generally possess the Alk enzyme system, which is involved in the metabolic pathway for the degradation of alkanes, the main compounds found in petroleum and its derivatives [3]. The functional Alk enzyme system comprises the transmembrane alkane monooxygenase AlkB (encoded by the *alkB* gene and involved in the initial activation step of aerobic aliphatic hydrocarbon metabolism) and two co-factors named rubredoxin (*alkF*) and rubredoxin reductase (*alkG*). These co-factors are responsible for transferring the electrons involved in alkane hydroxylation by AlkB [4]. In other studies, the *alkB* gene has been used as a biomarker for the determination of the abundance and diversity of alkane-degrading bacteria [5−7].

Bacteria that possess the Alk enzyme system are valuable in environmental bioremediation and biocatalysis for the synthesis of industrial compounds, including drugs, pravastatin, and other compounds [8]. The use of alkane-degrading bacteria in bioprocesses to produce valuable chemicals by transforming alkanes from hydrocarbon-contaminated samples is considered one of the most important biotechnological applications of these bacteria [9]. Therefore, many studies report the detection and further characterization of *alkB* genes in a wide variety of bacterial genera [4], [10], [11].

However, the *alkB* genes characterized thus far may only represent a small fraction of the diversity found in natural environments and a limited biotechnological potential of alkane-degrading bacteria. Kuhn and co-workers [5] found new *alkB* genes when contaminated and uncontaminated marine sediments in Admiralty Bay, King George Island, Antarctica were analyzed. Similar results were obtained for samples from the Timor Sea in Australia [12] and from chronically polluted, sub Antarctic coastal sediments [13].

Although all AlkB proteins share considerable sequence homology, the nucleotide sequences encoding the *alkB* gene vary widely within the *alkB*-containing bacteria. To overcome this limitation and study the *alkB* diversity in natural environments, different *alkB* primers have been described [5], [14], [15]. However, each set of primers is specific for primarily one group of bacteria [15−17], and designing broad-ranging *alkB* primers is not an easy task. Consequently, the presence and diversity of *alkB* sequences have likely been underestimated in the environmental samples previously studied.

In this study, we propose to use a combination of primers, rather than designing new primers, to improve the identification of the *alkB* diversity in different environments. For this purpose, we retrieved several pairs of *alkB*-targeting primers from the literature and tested their specificities against alkane-degrading bacteria previously isolated by our research group. These bacteria are representatives of Proteobacteria, Actinobacteria, Flavobacteria and Firmicutes groups. After selecting the combination of *alkB*-targeting primers showing the broadest coverage among the bacterial strains tested, we constructed *alkB* clone libraries using an oil-contaminated semiarid soil from Carmópolis, located in Sergipe (SE), Brazil to test the approach proposed here in the environment. Moreover, this strategy was also used to understand the *alkB* diversity in three pristine soil samples and one diesel-contaminated soil sample from King George Island, Antarctica. The data obtained suggest that the *alkB* diversity in soil environments may be higher than previously described.

## Materials and Methods

### *alkB*-targeting Primers

The list of *alkB*-targeting primer pairs used is presented in Table 1. The PCR reactions were performed under the conditions previously described for each pair of primers (Table 1).

**Table 1:** List of *alkB*-targeting primers used in this study.

Primer code^a^	*alkB*-targeting primers	Primer sequences	References	Approximate position of *alkB* fragments^b^
(a)	RHOSE	5′ ACG GSC AYT TCT ACR TCG 3′	[42]	481 to 823 nt^c^
	RHOAS	5′ CCG TAA RTG YTC GAG RTA G 3′		
(b)	Rh *alkB1*-F2	5′ ATC TGG GCG CGT TGG GAT TTG AGC G 3′	[43]	331 to 950 nt
	Rh *alkB1*-R1	5′ CGC ATG GTG ATC GCT GTG CCG CTG C 3′		
(c)	Rh *alkB2*-F2	5′ ACT TTG GCG CAG TCG TTT TAC GGC C 3′	[43]	462 to 1013 nt
	Rh *alkB2*-R1	5′ CCC ACT GGG TAG GTT GGG CGC ACC G 3′		
(d)	alkF	5′ GCI CAI AR ITI RKI CAY AA 3′	[5]	408 to 949 nt
	alkR	5′ GCI TGI TGI TCI SWR TGI CGY TG 3′		
(e)	alkB-1f	5′ AAY CAN GCN CAY GAR CTN GGN CAY AA 3′	[14]	402 to 949 nt
	alkB-1r	5′ GCR TGR TGR TCN GAR TGN CGY TG 3′		
(f)	alk-H1F	5′ CIG IIC ACG AII TIG GIC ACA AGA AGG 3′	[24]	406 to 950 nt
	alk-H3R	5′ IGC ITG ITG ATC III GTG ICG CTG IAG 3′		
(g)	AlkBF	5′ CCT GCT CCC GAT CCT CGA 3′	[44]	170 to 911 nt
	AlkBR	5′ TCG TAC CGC CCG CTG TCC AG 3′		
(h)	Alk-BFB	5′ GGT ACG GSC AYT TCT ACR TCG A 3′	[45]	477 to 956 nt
	Alk-BRB	5′ CGG RTT CGC GTG RTG RT 3′		

a Primer code used throughout the results section and figures.

b Reference position of the amplified fragment based on complete *alkB* gene sequence of *Pseudomonas putida* Gp01.

c nt = nucleotide.

Bacterial strains, test of bacterial growth using heptadecane as the sole carbon source and bacterial identification.

The bacterial strains used (Table 2) were isolated and described in previous studies [18−20]. The ability of these isolated strains to use heptadecane as the sole carbon source was determined as described by Alvarez et al. [19]. The 16S rRNA gene sequences were used for the identification of the bacterial strains. Genomic DNA was extracted using a protocol described by Pitcher et al. [21]. BOX-PCR was used to cluster the bacterial strains according to Versalovic et al. [22], and one representative strain from each BOX-PCR group was selected for 16S rRNA-based molecular identification. PCR amplification of the 16S rRNA coding gene and the molecular sequencing methodologies were performed as described in Alvarez et al. [19]. The partial 16S rRNA gene sequences (∼800 bp) were identified using the BLAST-N tool (blast.ncbi.nlm.nih.gov) on the National Center for Biotechnology Information (NCBI) website using the GenBank non-redundant database.

**Table 2:** PCR amplification of the *alkB* gene from alkane-degrading bacterial strains using different *alkB*-targeting primers.

			*alkB* primers^a^								
Strains	BLASTN identification	HEP	(a)	(b)	(c)	(d)	(e)	(f)	(g)	(h)	Reference or accession number (this study)
Ari_O 5A	γ^b^; *Acinetobacter baumannii*	+	−	−	−	+	+	−	−	+	KC715845
Ari_O 8	γ; *Acinetobacter baumannii*	+	−	−	−	+	+	−	−	+	KC715846
Br_lB 66	γ; *Acinetobacter baumannii*	+	−	−	−	+	−	−	−	+	KC715832
Br_lB 68	γ; *Acinetobacter baumannii*	+	−	−	−	+	−	−	+	+	KC715843
Bri_O 66	γ; *Acinetobacter baumannii*	+	−	−	−	+	−	−	−	+	KC715842
Cri_O 3	γ; *Acinetobacter baumannii*	+	−	−	−	+	+	+	−	+	KC715853
Ari_O 10	γ; *Acinetobacter calcoaceticus*	+	+	−	−	+	+	+	−	−	KC715847
Ari_O 20	γ; *Acinetobacter oleivorans*	+	−	−	−	+	−	−	−	−	KC715856
Ari_O 1	γ; *Enterobacter gergoviae*	+	−	+	−	+	−	−	−	−	[20]
Br_O 3B	γ; *Pseudomonas aeruginosa*	+	−	+	−	−	+	−	+	−	KC715836
Ar_lB 45B	γ; *Pseudomonas aeruginosa*	+	−	+	−	−	+	−	+	−	KC715837
Br_O 5A.1	γ; *Pseudomonas aeruginosa*	+	−	+	−	−	+	−	−	−	KC715841
Ar_lB 49	γ; *Pseudomonas* sp. Bu34	+	−	+	−	−	+	−	−	−	KC715839
Ar_lB 50B	γ; *Pseudomonas* sp. Bu34	+	−	+	−	−	+	−	+	−	KC715831
Br_lB N1B	γ; *Stenotrophomonas maltophilia*	+	−	−	+	−	−	+	−	+	KC715848
PBL 3.1	γ; *Stenotrophomonas* sp.	+	-	+	+	−	−	−	−	−	[19]
EM	β; *Burkholderia seminalis*	+	−	−	+	−	+	+	−	+	KC715855
Bri_O 42B	β; *Cupriavidus gilardii*	+	−	−	−	−	−	−	−	−	KC715844
Bri_O 51	β; *Cupriavidus* sp. C14	+	−	−	−	−	−	−	−	−	KC715830
Ar_lB N1	β; *Pandoraea* sp. KBA1SM3	+	−	+	−	−	−	+	−	−	[20]
Cr_lB N2B.1	α; *Agrobacterium tumefaciens*	+	−	−	−	−	+	−	+	+	KC715850
Cr_O 46.1	α; *Bosea minatitlanensis*	+	−	−	−	−	−	−	−	−	[20]
Cr_O 49.2	α; *Bosea minatitlanensis*	+	−	−	+	−	−	−	−	−	[20]
Ari_O 50	α; *Mycoplana bullata*	+	−	−	−	−	−	+	−	−	[20]
Cr_lB 49A	α; *Rhizobium* sp. JNVU TL9	+	−	−	−	−	−	−	−	−	KC715840
Cr_lB N4A	α; *Rhizobium* sp. VL-2	+	−	−	−	−	+	−	+	−	KC715852
Bri_O 61	Flavobacteria; *Chryseobacterium daecheongense*	+	−	−	−	+	−	−	−	+	KC715854
Cr_lB N2B.2	Firmicutes; *Bacillus cereus*	+	−	−	−	−	−	−	−	−	KC715851
Cr_lB 43	Firmicutes; *Bacillus cereus*	+	−	−	−	−	−	−	−	−	KC715838
P4	Actinomycetales; *Dietzia cinnamea*	+	−	−	−	−	+	+	−	+	[18]
Bri_O 50	Actinomycetales; *Gordonia amicalis*	+	+	−	−	−	+	+	+	−	[20]
Cr_O 47	Actinomycetales; *Gordonia amicalis*	+	−	−	−	−	+	+	−	−	[20]
LBOa 3.2	Actinomycetales; *Gordonia alkanivorans*	+	−	−	−	−	−	−	−	−	[19]
DTSB 2.5	Actinomycetales; *Gordonia rubriperctinta*	+	−	−	+	−	−	+	+	+	[19]
DLB 1.9	Actinomycetales; *Nocardia veterana*	+	+	−	−	−	−	+	−	−	[19]
Ari_O Alk	Actinomycetales; *Rhodococcus equi*	+	−	−	−	−	−	+	−	+	[20]
Bri_lB 51	Actinomycetales; *Rhodococcus equi*	+	+	−	−	−	+	+	−	+	KC715834
Cr_lB 46A	Actinomycetales; *Rhodococcus equi*	+	+	−	+	−	+	+	−	+	KC715833
Cr_lB 47B	Actinomycetales; *Rhodococcus equi*	+	−	−	+	−	+	+	−	−	KC715849
Cr_lB 93	Actinomycetales; *Rhodococcus equi*	+	+	−	+	−	+	+	−	+	KC715835
DLB 1.4	Actinomycetales; *Rhodococcus equi*	+	−	−	−	−	−	+	−	+	[19]
DLB 3.4	Actinomycetales; *Rhodococcus* sp. PA	+	+	−	−	−	+	+	−	+	[19]
DTSB 3.5	Actinomycetales; *Rhodococcus* sp. DASAN	+	+	−	+	−	+	+	−	+	[19]
Number of strains (%) amplified by each *alkB*-targeting primer			18.6%	18.6%	20.9%	23.3%	48.8%	44.2%	18.6%	44.2%	

a The codes of *alkB*-targeting primers are those described in Table 1.

b Phylogenetic position of isolated alkane-degrading bacterial strains; α, Alphaproteobacteria; β, Betaproteobacteria; γ, Gammaproteobacteria.

HEP – all strains were able to grow with heptadecane as the sole carbon source.

### Selection and Combined Use of *alkB*-targeting Primers to Study the *alkB* Diversity in Different Soils

The *alkB*-targeting primers were chosen based on the combination of primers that allowed for the detection of the *alkB* gene in as many bacterial strains tested as possible. Primers were combined manually in pairs and triplets, and the number of bacterial strains detected by the combination of primers used was determined.

To test the approach suggested here (the use of a combination of *alkB*-targeting primers) to better describe the *alkB* diversity in soil environments, we constructed clone libraries using individually the chosen primers and the DNA extracted from contaminated soil samples obtained in Carmópolis (denoted as sC throughout the manuscript), SE, Brazil, where the majority of the bacterial strains presented in Table 2 were isolated [18−20]. Furthermore, this strategy was extended to study the *alkB* diversity present in diesel-contaminated soil (denoted as s3) and three uncontaminated (pristine) soil samples (sY, sI and sR) from King George Island, Antarctica (Fig. S1). These Antarctic soils were chosen because previous studies have suggested that the soils may contain an underestimated diversity of genes coding for AlkB enzymes or even new *alkB* coding genes [20], [23]. Moreover, the soil samples were selected based on their different chemical and physicochemical properties (Table 3). All samplings were performed in triplicate, and the soil samples were kept at −20°C until DNA extraction. All necessary permits were obtained for the soil samplings through the research projects funded by Petrobras (Carmópolis soil) and the Brazilian Antarctic Program (Antarctic soils).

**Table 3:** Chemical and physicochemical properties of the soils from Carmópolis, Brazil and King George Island in Maritime Antarctica^a^.

Sample	pH	P	K	Ca	Mg	H+Al	S	OM	TPH
		mg dm^−3^		cmol_c_ dm^−3^				dag kg^−1^	mg g^−1^
sC	6.0	0.34	nd^b^	0.52	0.47	nd	nd	5.8	16,000
s3	7.0	181	79	3.6	1.58	0.8	5.4	1.6	20,619
sY	3.6	514	40	10.0	5.5	31.9	15.8	0.45	UDL^c^
sI	5.7	786	9	11.0	6.0	11.0	17.8	5.11	UDL
sR	4.3	1005	9	12.0	7.0	20.0	19.5	0.97	UDL

a data from Jurelevicius et al. [20], [23] and this study.

b not determined.

c under the detection limit of the method used.

### DNA Extraction and Clone Library Construction

Total DNA was extracted directly from 0.5 g of soil using the Fast DNA Spin Kit for soil (QBIOgene, Carlsbad, CA) following the manufacturer’s instructions. To improve the coverage of the results obtained here, DNA extractions and PCR reactions were performed in triplicate for each sampling site.

To construct the clone libraries, the fragments of the *alkB* gene were PCR amplified using DNA from the soils described above and with the primers (f), (e) and (d) described by Chénier et al. [24], Kloos et al. [14] and Kuhn et al. [5], respectively (Table 1). PCR amplification followed the conditions previously described for each pair of primers (Table 1), and the 25 µl-PCR reaction mix comprised 1 µl of template DNA (30–50 ng), 0.5 pmol of primers, 0.2 mM of each dNTP, 5 µl of 5X PCR buffer (100 mM Tris-HCl, pH 9.0, and 500 mM KCl), 1.5 mM MgCl_2_ and 1 U of *Taq* DNA polymerase (Promega, Madison, WI, USA).

The PCR amplification products were used to construct the clone libraries. Before cloning procedures, the PCR products obtained from the different triplicates of each sampling site were pooled and purified using the Wizard SV Gel and PCR Clean-up System (Promega). Purified amplicons were then cloned using the InsTAclone PCR Cloning Kit (Fermentas, Maryland, USA) following the supplier’s instructions. The insert-containing clones were sequenced using the forward primer M13F (5′-GTA AAA CGA CGG CCA GT-3′) in the vector pTZ57R/T on an ABI Prism 3100 automatic sequencer (Applied Biosystems Inc., CA, USA) using Macrogen (South Korea) facilities.

### Sequence Analysis

The electropherogram files generated by sequencing were analyzed using the Phred program [25] for base calling and trimming of vector and low-quality (<20) sequences. Vector contamination and primer sequences were removed manually using Bioedit software (Ibis Biosciences Inc., CA, USA). To analyze only the overlapping fragments of the *alkB* gene amplified from all pairs of primer used, the obtained sequences were aligned using the package software Clustal X [26] and edited using Bioedit. Only the overlapping regions of *alkB* fragments were used in the following steps. The MOTHUR software [27] was used to classify the *alkB* genes into operational taxonomic units (OTUs) with 97% similarity. Next, the OTU-generated matrices were used to calculate the species richness using Chao1 estimators [28] and the Shannon-Weaver diversity index [29]. Coverage (C) was also calculated, where C equals 1−*n*1/N, and *n*1/N is the ratio of clones that appeared only once (*n*1) to the total number of clones (N) [30]. Boneh’s estimator was used to estimate the number of additional OTUs that would be observed if an additional sampling of clones would have been performed [31]. Finally, the diversity of OTUs and those found in common after the amplification with the different primers used were examined using rarefaction analysis and Venn diagrams.

### Richness of *alkB* Phylotypes

The increase in *alkB* richness as a result of the use of the combination of *alkB*-targeting primers was calculated based on Venn diagram results. The calculation was performed as follows: (lR1+lR2)/hR, where the hR corresponds to the richness of *alkB* phylotypes detected only in the richest clone library, and (lR1+ lR2) represents the sum of the richness of *alkB* phylotypes observed in the other libraries (but not in hR).

### Phylogenetic Analyses of *alkB* Phylotypes

The representatives of each OTU (at a distance level of 3%) obtained from the analyses of the clone libraries were taxonomically assigned using the BLAST-n tool on the NCBI website and the GenBank non-redundant database. A phylogenetic tree was constructed with representatives of each OTU found within the libraries and with closely related sequences that were recovered from the GenBank database. Sequence alignment was performed by Clustal X software [26], and the aligned sequences were then used to construct the phylogenetic tree using the neighbor-joining method in the MEGA 5 software [32]. Bootstrap analyses were performed with 1,000 repetitions, and only values higher than 99% are shown in the phylogenetic tree.

The nucleotide sequences were translated using the Transeq tool (http://www.ebi.ac.uk/Tools/emboss/transeq/) on the European Bioinformatics Institute website, and deduced amino acids were directly compared with the Protein Database using the algorithm BLASTP from NCBI to check if the sequenced *alkB* genes possess the conserved motifs common to AlkB proteins.

### Clone Library Comparisons

The structure of the *alkB* phylotypes in each library was compared. For the comparison, a dendrogram describing the dissimilarity (1-similarity) among the clone libraries from each sampling site was clustered using the UPGMA algorithm and the Jaccard similarity coefficient based on the observed richness. To perform UniFrac-based library comparisons [33], neighborhood-joining trees were constructed using the MEGA 5 software [32]. An unweighted UniFrac significance test was used to estimate whether the clone libraries corresponding to each soil sample were significantly different. One hundred permutations were performed, and the P-values were corrected for multiple comparisons using the Bonferroni correction [34].

### Nucleotide Sequence Accession Numbers

The sequences obtained from the clone libraries were deposited in the GenBank database with the following accession numbers: KC733460–KC733713.

## Results

### Growth and Identification of the Bacterial Strains Using Heptadecane as the Sole Carbon Source

From 85 oil-degrading bacteria previously described in von der Weid et al. [18], Alvarez et al. [19] and Jurelevicius et al. [20], 64 were able to use heptadecane as the sole carbon source (an example is shown in Fig. S2). BOX-PCR clustered these bacteria into 43 different groups (data not shown), and a representative strain of each BOX group was further identified through 16S rRNA gene sequencing (Table 2). The results showed that the bacterial strains are distributed amongst Actinomycetales, Firmicutes, Flavobacteria and Alpha, Beta and Gamma proteobacteria (Table 2).

### Selection of the *alkB*-targeting Primers

The amplification range of eight different *alkB*-targeting primers previously described (Table 1) was determined by direct PCR amplification using the DNA of the alkane-degrading bacterial strains listed in Table 2. The use of each primer pair resulted in different amplification patterns. Using primers (a), (b), (c) and (g), only 18.6 to 20.9% of the bacterial strains tested were amplified. The broadest range of amplification (48.8% of the strains tested) was observed with primer pair (e), and primers (h) and (f) produced amplification products in 44.2% of the strains tested (Table 2). Primer pair (d) resulted in the amplification of the *alkB* gene in 23.3% of the strains tested, and most of these strains were identified as *Acinetobacter* (Table 2). However, the results showed that none of the primers used were highly specific to one particular phylogenetic group. PCR amplification also varied among strains from the same species, depending on the set of primers used (Table 2). Finally, the *alkB* gene was not detected in 7 (16.3%) of the alkane-degrading bacterial strains tested. These strains belong to the genera *Cupriavidus*, *Bacillus*, *Gordonia*, *Bosea* and *Rhizobium* (Table 2).

To enhance the detection of the *alkB* gene in previously isolated alkane-degrading bacteria, the *alkB*-targeting primers were combined into pairs (28 combinations) or triplets (56 combinations). The combination of primers selected allowed for the detection of the *alkB* gene in as many bacterial strains tested as possible (Table 2). Therefore, primers (d), (e) and (f) were chosen as they covered 79% of the alkane-degrading bacterial strains tested. The addition of a fourth pair of primers did not improve the detection range achieved with the use of the three sets of primers (Table 2).

### Validation of the Use of the Selected *alkB*-targeting Primers to Study the *alkB* Diversity in Soil DNA Samples

The three selected *alkB*-targeting primers were used to amplify *alkB* genes present in an oil-contaminated soil sample from Carmópolis, SE, Brazil, which was the source of the majority of the strains tested. Additionally, the same strategy was used to amplify *alkB* genes in one diesel-contaminated site and three pristine soil samples from King George Island, Antarctica. *alkB* genes were detected in all sampling sites, except for site sY when primer pair (d) was used.

**Figure 1:**
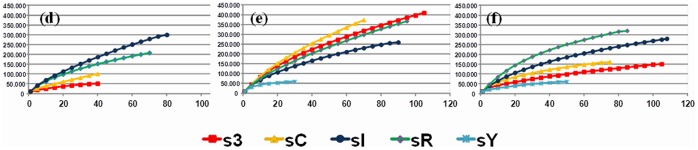
Rarefaction analysis of *alkB* clone libraries using a distance level of 97% similarity. The clone libraries are denoted as follows: the letters (d), (e) and (f) correspond to the *alkB*-targeting primers described in Table 1, and sI, sR, sY, s3 and sC are the sampling site codes (described in Materials and Methods).

Clone libraries were used to describe the *alkB* phylotypes that resulted from the amplification by each pair of primers. Only the overlapping regions of *alkB* fragments amplified by the primers chosen were selected and used. The *alkB* genes were clustered into OTU groups (referred to as *alkB* phylotypes) that were defined as sequences with more than 97% similarity. Rarefaction curves indicated that the number of clones screened from all soil samples was sufficient to reveal the majority of *alkB* phylotypes within the community because the estimates of sequence types tended to reach a plateau using 97% sequence identity (Fig. 1). The data obtained from the statistical analyses showed that the clone libraries covered 65 to 95% of the *alkB* phylotypes that resulted from the PCR amplification using each pair of primers and the different soil samples (Table 4). Boneh’s estimator showed that a new sequencing effort would increase the *alkB* phylotypes to a maximum of nine new *alkB* phylotypes (Table 4). Considering the results obtained from the sC sample, the highest richness and diversity of *alkB* phylotypes were detected by using primer pair (e) (Table 4). Considering the soil sampling sites s3 and sR from King George Island, the results also showed that the highest richness of *alkB* phylotypes was observed in the clone libraries constructed with PCR amplification products obtained using primer pair (e), followed by the use of (f) and (d) (Table 4). In the sampling site sI, the highest richness was observed in the clone libraries constructed with PCR amplification products obtained with the use of primer (d), followed by those from primers (f) and (e); the highest richness of *alkB* phylotypes in sampling site sY was observed with the use of primer pair (f) (Table 4).

**Table 4:** Data obtained from the statistical analyses of clone libraries.

	uncontaminated sampling sites								contaminated sampling sites					
	d_sI^a^	e_sI	f_sI	d_sR	e_sR	f_sR	e_sY	f_sY	d_s3	e_s3	f_s3	d_sC	e_sC	f_sC
Sobs	30	26	28	21	37	32	6	6	5	42	15	10	38	16
*Chao1* b	57.14	35.75	46.20	30.17	79.86	62.00	7	7.50	5.33	129.75	33	38	80.86	21.25
*Chao* (lower bound)^c^	39.04	28.67	33.10	23.29	52.42	40.72	6.07	6.15	5.02	71.88	18.98	17.47	53.42	17.00
*Chao* (upper bound)^c^	111.51	61.54	92.89	57.66	156.10	135.19	19.66	21.08	10.96	299.73	96.31	114.95	157.10	43.61
*ACE* d	198.41	78.81	47.16	56.20	226.44	51.53	7.48	14.69	7	167.31	46.03	176.93	155.29	34.62
Coverage	0.74	0.85	0.87	0.85	0.74	0.80	0.93	0.94	0.95	0.75	0.91	0.79	0.65	0.90
Shannon^e^	2.70	2.60	2.53	2.37	2.96	3.18	1.43	0.81	0.65	3.26	1.61	1.29	3.34	2.11
Boneh`s^f^	6.26	4.68	4.62	3.79	7.53	5.06	0.57	0.94	0.86	7.81	2.56	2.06	7.69	2.46

a The clone libraries are denoted as: letters d, e and f (representing the *alkB*-targeting primers as described in Table 1), followed by the sampling site codes sI, sR, sY (three uncontaminated (pristine) soil samples) and s3 (one diesel contaminated soil sample) from King George Island, Antarctica (Fig. S1) and sC (oil-contaminated soil) from Carmópolis.

b Species richness [28];

c Confidence intervals [46];

d Species richness [47];

e Shannon’s diversity index (H’) [29];

f Boneh estimator [31].

### Improved Detection of the Richness of *alkB* Phylotypes

**Figure 2:**
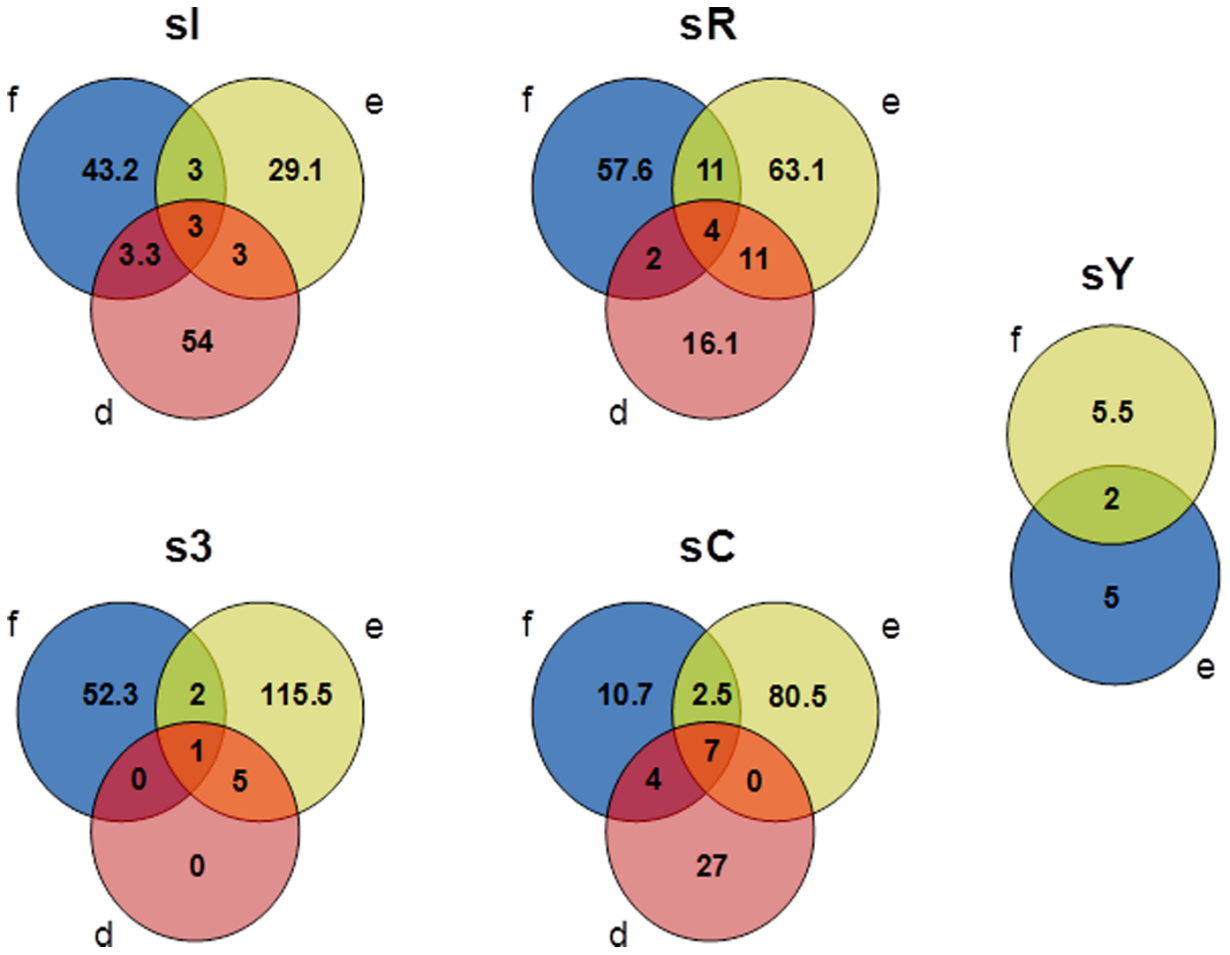
A Venn diagram of *alkB* clone libraries using a distance level of 97% similarity. The numbers represent the richness and the shared richness of each library using the Chao richness index [28]. The Venn diagram groups are denoted as follows: sI, sR, sY, s3 and sC represent the sampling sites, and the letters (d), (e) and (f) correspond to the *alkB*-targeting primers used in the PCR amplification of the *alkB* gene, as described in Table 2.

Chao richness-based Venn diagrams showed the shared presence of *alkB* phylotypes detected by each *alkB*-targeting primer and from each sampling site (Fig. 2). The results showed that the clone libraries generated by each primer pair shared low numbers of common *alkB* phylotypes (Fig. 2). Subsequently, the results from the Venn diagrams were used to calculate the gain in *alkB* phylotype richness by using the combined *alkB*-targeting primer strategy. The use of this strategy to describe the *alkB* diversity in sC soil resulted in a 47% increase in *alkB* richness (Fig. 2, Fig. S3). The same strategy used to study the *alkB* diversity in soils from King George Island, Antarctica, resulted in an increase of 45 to 139% in *alkB* richness (Fig. 2, Fig. S3).

### Phylogenetic Analyses of *alkB* Genes

A total of 254 OTUs (referred to as *alkB* phylotypes) were obtained in this study. The phylogenetic analyses of the *alkB* phylotypes showed the detection of sequences sharing 64 to 100% identity with *alkB* sequences previously deposited in GenBank. However, from the 254 *alkB* phylotypes detected here, only 20% of the sequences showed identities 90% or higher with known sequences (35% of the sequences shared identities of 80% or lower with known sequences). Nucleotide translating analysis followed by deduced amino acid analysis using BLAST-P tools showed that only one *alkB* phylotype obtained did not encode a potentially functional AlkB. A stop codon was detected in the nucleotide sequence of this phylotype, and consequently this phylotype was excluded from further analyses. All other *alkB* phylotypes had conserved amino acid motifs found in functional AlkB enzymes.

**Figure 3:**
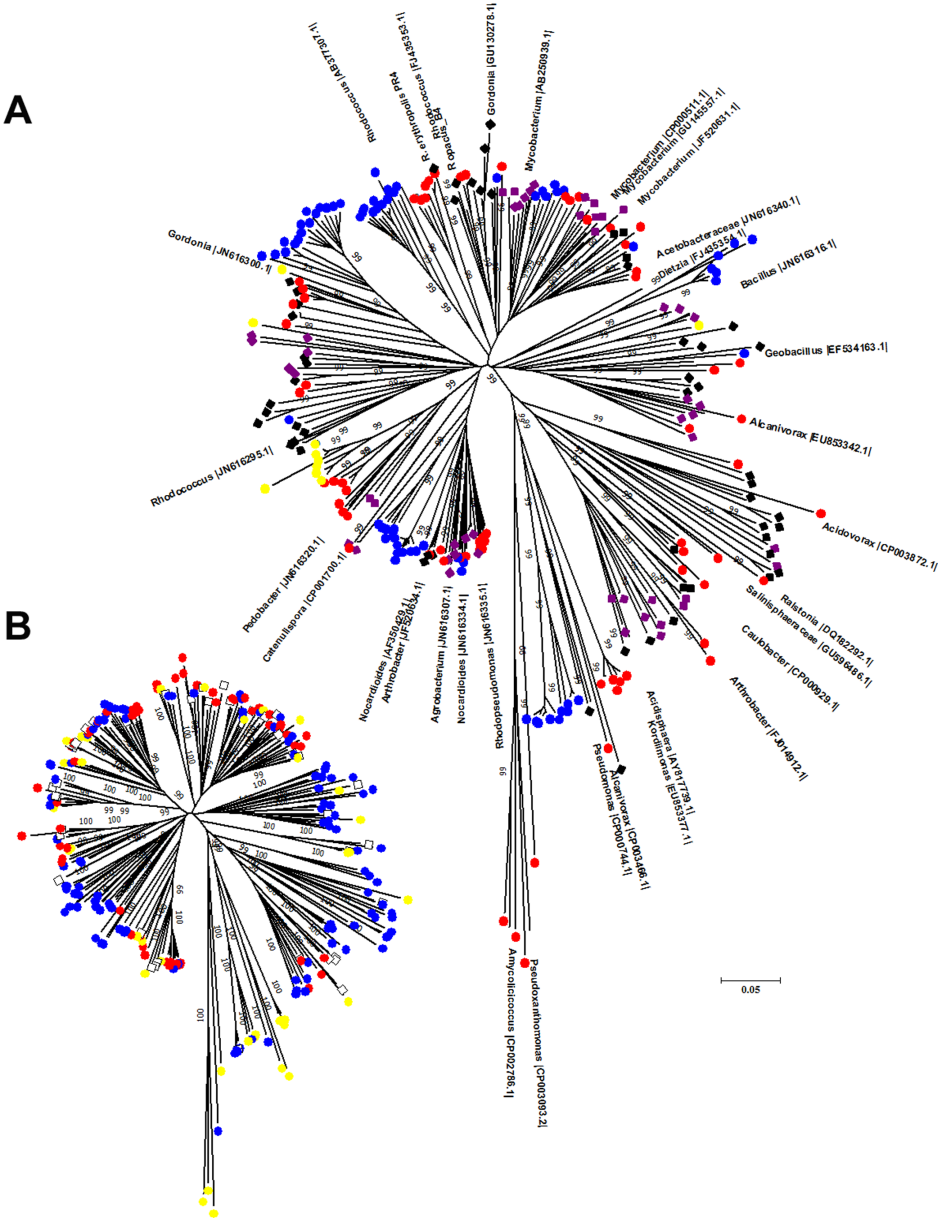
Phylogenetic tree of the *alkB* sequences obtained from sI, sR, sY, s3 and sC soil libraries and from the closely related *alkB* genes from the GenBank database (A). The tree was constructed using the neighbor-joining (NJ) method and MEGA 5 software. The corresponding colors for the different sampling sites are: red (sI), blue (sR), yellow (sY), black (s3) and purple (sC). (B) The same phylogenetic tree showing the distribution of the *alkB* phylotypes resulting from the amplification with the different targeting primers. The colors corresponding to the primers used are: yellow - (d), blue - (e) and red - (f). The color white was used for phylotypes that originated from the PCR amplification with more than one primer.

Phylogenetic analyses of the *alkB* phylotypes showed that each of the *alkB*-targeting primers chosen was not specific to any monophyletic group of *alkB* genes because the primers were able to anneal to *alkB* gene sequences from diverse phylogenetic groups (Fig. 3A, B). In sampling site sC, the most representative *alkB* phylotypes (corresponding to 30% and 13.4% of the clone libraries) were related to *alkB* from *Mycobacterium chubuense* NBB4 (with 92% sequence identity) and to *alkB* from *Acidisphaera* sp. C197 (81% identity), respectively.

From the diesel-contaminated soil (s3) sampled in King George Island, the most abundant *alkB* phylotypes (29.7%) shared high identity (99%) with the *alkB* gene described in *Rhodococcus* sp. 28/19, an oil-degrading strain previously isolated from soils in Antarctica. Other *alkB* phylotypes obtained from the s3 sampling site were related to an *alkB* gene from non-cultivated bacteria detected in Arctic contaminated soils (99% identity) and to *alkB* described in a strain from the Acetobacteraceae family (89% identity). These *alkB* phylotypes correspond to 14.8% and 12.9% of the s3 clone library, respectively (Fig. 3A).

From the uncontaminated soils sampled in King George Island, most of the *alkB* phylotypes detected at sampling site sY (corresponding to 73.1% of the clones) were related to an *alkB* gene described in *Pedobacter* sp. MS245e (78 to 85% identity). Additionally, phylotypes related to *alkB* from *Bacillus* sp. MS238f (88% identity) corresponded to 23.3% of the sY clone library. In sampling site sI, the most abundant *alkB* phylotype (20.7%) shared 87% identity with *alkB* described in *Rhodococcus opacus* B4. Other phylotypes were associated with *alkB* genes from *Nocardia brasiliensis* ATCC 700358 (79% identity) and *Acidovorax* sp. KKS102 (73% identity), representing 13.6% and 9.5%, respectively, of the *alkB* phylotypes found at sampling site sI. In sample site sR, the most abundant *alkB* phylotype (15.1%) was related to an *alkB* gene from *Rhodococcus erythropolis* (77% identity). Other *alkB* phylotypes previously found in *Pseudomonas aeruginosa* PG201 and *Pedobacter* sp. MS245e represented 10.8% and 8.9%, respectively, of the clone library obtained from sampling site sR (Fig. 3A).

### Comparison among Libraries

**Figure 4:**
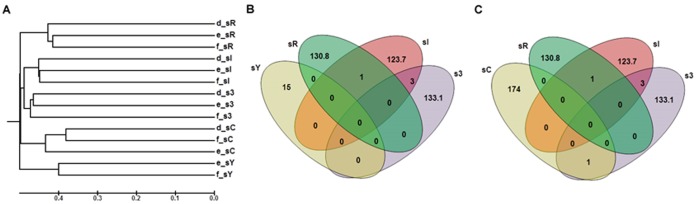
Dendrogram describing the dissimilarity (1-similarity) among the sampling sites (A). The groups were clustered using the UPGMA algorithm and the Jaccard similarity coefficient based on the observed richness. The clone libraries are denoted as follows: the letters (d), (e) and (f) correspond to the *alkB*-targeting primers described in Table 2, and the sampling sites (sI, sR, sY, s3 and sC) are described in the Materials and Methods. (B) and (C) Venn diagrams of all *alkB* phylotypes detected in each sampling site using a distance level of 97% similarity.

The *alkB* diversity was used to further analyze the structure of alkane-degrading bacteria present in each sampling site. In all cases, the different soil samples were clustered separately in the dendrogram analyses with less than 40% similarity (Fig. 4). Low numbers of *alkB* phylotypes were shared between two different sampling sites, and common *alkB* phylotypes were not found in more than two sampling sites (Table S1, Fig. 4 B, C). For example, the *alkB* phylotypes shared among sampling sites s3 and sI were related to an *alkB* gene from the Acetobacteraceae family (89% sequence identity) and represented only 12.9% and 2%, respectively, of the phylotypes found in these soils. Finally, qualitative beta-diversity measures using unweighted UniFrac analysis confirmed the differences observed among the *alkB* clone libraries obtained by each sampling point studied here (*p*<0.001).

## Discussion

The importance of the alkane monooxygenase AlkB in bioremediation processes of hydrocarbon-contaminated environments and in biocatalysis for the production of useful compounds justifies the worldwide interest in the distribution of the *alkB* gene in different environments [3], [35]. However, the current literature shows that the diversity of the *alkB* gene in environmental samples is still far from being well characterized [5], [6], [13], [14].

In this study, we show that the use of a single pair of primers for the PCR amplification of the *alkB* gene in soil environments as well as in isolated alkane-degrading bacteria limits the range of detection of this gene. However, the use of a combination of *alkB*-targeting primers results in a more reliable detection of the *alkB* gene in alkane-degrading bacteria from different soil samples. Even with considering the best combination of *alkB*-targeting primers, the presence of *alkB* genes was not detected in 21% of the bacterial strains tested. In a few of these strains, homologous *alkB* sequences have been previously detected by dot blot hybridization [20], [36]. Therefore, enzymes related to cytochrome P450 or others [10], [35] could be responsible for alkane degradation in the remaining strains. Nevertheless, if only the total number (79%) of strains detected with the combination of *alkB*-targeting primers is considered, our results indicate that 30.2% or more of *alkB*-possessing bacteria would be missed from environmental analyses with the use of individual *alkB*-targeting primers.

The use of different *alkB*-targeting primers to detect *alkB* genes in different bacterial isolates and also in environmental samples had been used before through dot blot hybridization and qPCR analyses [1], [6], [16], [37−40]. However, no studies have focused on the coverage of each primer and the benefit of using the different primers pairs to increase the diversity and richness of *alkB* phylotypes. Our results demonstrated that the use of a combination of *alkB*-targeting primers resulted in up to 139% increase in the richness of *alkB* gene phylotypes obtained in the soils used here. Moreover, the analysis of the clone libraries showed that the richness of *alkB* phylotypes resulting from the amplification using each of the chosen primers was dependent on the sampling site. Although most of our results indicated that primer pair (e) designed by Kloos et al. [14] generated the most diverse clone libraries in the samples studied, the use of primer pair (d) [5] resulted in the highest diversity and richness at sampling site sI. In contrast, the lowest diversity and richness were detected at this site using primer pair (e). The chemical and physicochemical properties of the soils, such as pH, TPH, organic matter and/or plant litter, may have influenced the diversity and richness of *alkB* phylotypes, as suggested by other studies [17], [23], [39], [41].

Although the sampling sites used in this study have been studied before [20], [23], this study is the first to perform a broader *alkB* sequencing analysis in these soils. Interestingly, our results suggest that different *alkB* phylotypes were selected depending on the sampling site analyzed, and no phylotype was shared between more than two different King George Island sampling sites (Fig. 4 B, C). In contrast with the results obtained by Powell et al. [17] who studied the alkane-degrading bacteria present on sub-Antarctic Macquarie Island, the sampling sites used here (with their own chemical and physicochemical properties) were sufficient to determine the *alkB*-containing bacterial community structure (Table 3, Fig. 4 A, B, C). The observation that *alkB* clone libraries obtained from each sampling point were considerably different from each other (Fig. 4A) in addition to the results obtained from the qualitative beta-diversity measures UniFrac analysis (*p*<0.001) corroborate the above statement.

The low identities observed among the *alkB* genes from the different environments studied here (Fig. 3) may indicate an *alkB* gene diversity yet uncharacterized in natural environments, as suggested previously [5], [6], [13], [14], [23]. Also, the low specificity of the primers used in this study for any bacterial phylogenetic group may be explained by the occurrence of horizontal gene transfer among *alkB*-containing bacteria [10]. Finally, the *alkB* phylotypes detected here possess conserved amino acid motifs present in functional AlkB enzymes, suggesting their functionality in natural environments. As the properties of the enzymes encoded by these *alkB* genes are still uncharacterized in soils from Carmópolis and Antarctica, the isolation and characterization of bacteria harboring these genes are still necessary.

## Supporting Information

Figure S1 **Map of Antarctic continent adapted from Jurelevicius et al.** [**23**] **.** King George Island, the biggest island of the South Shetland archipelago, is shown together with the sample sites (indicated by arrows).
(TIF)

Figure S2 **Growth of some isolated strains using heptadecane as the sole carbon source.** The columns represent different bacterial strains (1 to 5 - strains Br_O 3B, Cri_O 3, Ar_lB 45B, Bri_O 51 and Ar_lB 50B, respectively, and 6 - negative control), and the rows represent (A) the negative control where the strains were inoculated in mineral medium (Bushnell Haas) and (B) the strains were inoculated in mineral medium added with heptadecane (0.1% v/v) as the sole carbon source.
(TIF)

Figure S3 **ichness of** ***alkB*** **phylotypes observed in each clone library and also in all clone libraries (sum) of each sampling site.** The clone libraries are denoted as follows: the letters d, e and f correspond to the *alkB*-targeting primers as described in Table 1 and sI, sR, sY, s3 and sC correspond to the sampling sites as described in Materials and Methods.
(TIF)

Table S1 **Shared** ***alkB*** **phylotypes among the different clone libraries.**
(DOCX)
