# Supplementary figures and images for: The Use of a Combination of alkB Primers to Better Characterize the Distribution of Alkane-Degrading Bacteria
Source: PLoS One. 2013 Jun 18;8(6):e66565. doi: 10.1371/journal.pone.0066565 (PMC3688950; doi:10.1371/journal.pone.0066565)

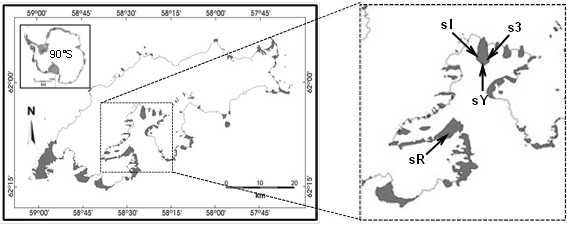

Supplement: Figure S1 — Map of Antarctic continent adapted from Jurelevicius et al. [23] . King George Island, the biggest island of the South Shetland archipelago, is shown together with the sample sites (indicated by arrows). (TIF) [file pone.0066565.s001.tif]

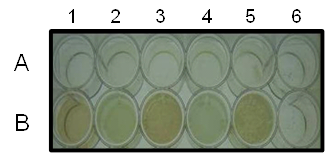

Supplement: Figure S2 — Growth of some isolated strains using heptadecane as the sole carbon source. The columns represent different bacterial strains (1 to 5 - strains Br_O 3B, Cri_O 3, Ar_lB 45B, Bri_O 51 and Ar_lB 50B, respectively, and 6 - negative control), and the rows represent (A) the negative control where the strains were inoculated in mineral medium (Bushnell Haas) and (B) the strains were inoculated in mineral medium added with heptadecane (0.1% v/v) as the sole carbon source. (TIF) [file pone.0066565.s002.tif]

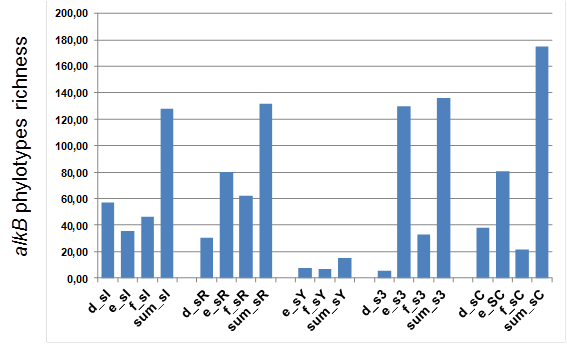

Supplement: Figure S3 — ichness of alkB phylotypes observed in each clone library and also in all clone libraries (sum) of each sampling site. The clone libraries are denoted as follows: the letters d, e and f correspond to the alkB-targeting primers as described in Table 1 and sI, sR, sY, s3 and sC correspond to the sampling sites as described in Materials and Methods. (TIF) [file pone.0066565.s003.tif]
