# Supplementary material for: The Use of a Combination of alkB Primers to Better Characterize the Distribution of Alkane-Degrading Bacteria
Source: PLoS One. 2013 Jun 18;8(6):e66565. doi: 10.1371/journal.pone.0066565 (PMC3688950; doi:10.1371/journal.pone.0066565)
Supplement: Table S1 — Shared alkB phylotypes among the different clone libraries. (DOCX) [file pone.0066565.s004.docx]

Table S1

|  | e_sI | d_sI | f_sI | e_sR | d_sR | f_sR | e_sY | f_sY | e_s3 | d_s3 | f_s3 | e_sC | d_sC | f_sC |
| --- | --- | --- | --- | --- | --- | --- | --- | --- | --- | --- | --- | --- | --- | --- |
| e_sI |  | 5.000 | 5.000 | 0.000 | 0.000 | 0.000 | 0.000 | 0.000 | 0.000 | 0.000 | 1.000 | 0.000 | 0.000 | 0.000 |
| d_sI |  |  | 5.000 | 1.000 | 1.000 | 1.000 | 0.000 | 0.000 | 1.000 | 1.000 | 1.000 | 0.000 | 0.000 | 0.000 |
| f_sI |  |  |  | 0.000 | 0.000 | 0.000 | 0.000 | 0.000 | 1.000 | 0.000 | 2.000 | 0.000 | 0.000 | 0.000 |
| e_sR |  |  |  |  | 8.000 | 10.000 | 0.000 | 0.000 | 0.000 | 0.000 | 0.000 | 0.000 | 0.000 | 0.000 |
| d_sR |  |  |  |  |  | 6.000 | 0.000 | 0.000 | 0.000 | 0.000 | 0.000 | 0.000 | 0.000 | 0.000 |
| f_sR |  |  |  |  |  |  | 0.000 | 0.000 | 0.000 | 0.000 | 0.000 | 0.000 | 0.000 | 0.000 |
| e_sY |  |  |  |  |  |  |  | 2.000 | 0.000 | 0.000 | 0.000 | 0.000 | 0.000 | 0.000 |
| f_sY |  |  |  |  |  |  |  |  | 0.000 | 0.000 | 0.000 | 0.000 | 0.000 | 0.000 |
| e_s3 |  |  |  |  |  |  |  |  |  | 3.000 | 3.000 | 0.000 | 0.000 | 0.000 |
| d_s3 |  |  |  |  |  |  |  |  |  |  | 1.000 | 0.000 | 0.000 | 0.000 |
| f_s3 |  |  |  |  |  |  |  |  |  |  |  | 0.000 | 1.000 | 1.000 |
| e_sC |  |  |  |  |  |  |  |  |  |  |  |  | 4.000 | 8.000 |
| d_sC |  |  |  |  |  |  |  |  |  |  |  |  |  | 5.000 |
| f_sC |  |  |  |  |  |  |  |  |  |  |  |  |  |  |
